# Monte Carlo Simulations for the Detection of Buried Objects Using Single Sided Backscattered Radiation

**DOI:** 10.1371/journal.pone.0135769

**Published:** 2015-09-08

**Authors:** Mary Yip, M. Iqbal Saripan, Kevin Wells, David A. Bradley

**Affiliations:** 1 Centre for Vision, Speech and Signal Processing, Faculty of Engineering and Physical Sciences, University of Surrey, Guildford, GU2 7XH, United Kingdom; 2 Department of Physics, Faculty of Engineering and Physical Sciences, University of Surrey, Guildford, Surrey, GU2 7XH, United Kingdom; 3 Department of Computer and Communication Systems Engineering, Faculty of Engineering, Universiti Putra Malaysia (UPM), 43400 UPM, Selangor, Malaysia; Oregon State University, UNITED STATES

## Abstract

**Background:**

Detection of buried improvised explosive devices (IEDs) is a delicate task, leading to a need to develop sensitive stand-off detection technology. The shape, composition and size of the IEDs can be expected to be revised over time in an effort to overcome increasingly sophisticated detection methods. As an example, for the most part, landmines are found through metal detection which has led to increasing use of non-ferrous materials such as wood or plastic containers for chemical based explosives being developed.

**Methodology:**

Monte Carlo simulations have been undertaken considering three different commercially available detector materials (hyperpure-Ge (HPGe), lanthanum(III) bromide (LaBr) and thallium activated sodium iodide (NaI(Tl)), applied at a stand-off distance of 50 cm from the surface and burial depths of 0, 5 and 10 cm, with sand as the obfuscating medium. Target materials representing medium density wood and mild steel have been considered. Each detector has been modelled as a 10 cm thick cylinder with a 20 cm diameter.

**Principal Findings:**

It appears that HPGe represents the most promising detector for this application. Although it was not the highest density material studied, its excellent energy resolving capability leads to the highest quality spectra from which detection decisions can be inferred.

**Conclusions:**

The simulation work undertaken here suggests that a vehicle-born threat detection system could be envisaged using a single betatron and a series of detectors operating in parallel observing the space directly in front of the vehicle path. Furthermore, results show that non-ferrous materials such as wood can be effectively discerned in such remote-operated detection system, with the potential to apply a signature analysis template matching technique for real-time analysis of such data.

## Introduction

Detection of buried improvised explosive devices (IEDs) is a delicate task, leading to a need to develop sensitive stand-off detection technology. Not least among the challenges in producing high specificity (ruling out the presence of a threat in the absence of a threat), high sensitivity (ruling in the presence of a threat in the presence of a threat) technology is that the shape, composition and size of the IEDs can be expected to be revised over time in an effort to overcome increasingly sophisticated detection methods. As an example, for the most part, landmines are found through metal detection which has led to increasing use of non-ferrous materials such as wood, plastic containers for chemical based explosives being developed [1][2], [3].

Single-sided backscatter techniques offer the advantage that they may be used in a variety of detection scenarios, unlike transmission-based techniques which require that the source and detector be on opposite sides of the object. Furthermore, landmine detection methods have followed one of two routes: neutron or photon techniques. Neutron based methods are generally used to identify explosives by their stoichiometry [4]. This is an advantage where landmines and IEDs are increasingly based on non-metallic componentry e.g. plastic, wood and/or chemical componentry. However, neutron techniques are susceptible to high false positives (having a likely desensitizing effect upon the operator) due to soil moisture variations, surface irregularities and detection height variations. On the other hand, since these approaches yield low doses to the user, it is often seen as an advantage in terms of developing handheld devices [1].

Photon techniques involve firing a beam of X- or gamma-ray photons towards an area of interest and detecting the return photon flux. While such backscattered radiography has been suggested to offer an effective imaging solution [5], it nevertheless largely depends upon human interpretation of the data. Signature analysis of the return signal [6] may offer a solution to this problem. Herein, we have investigated a method using penetrating radiation to induce positron annihilation (the so-called PIPAR method, described by [7]) to detect buried objects in sand. In this new work, we investigate a backscatter approach using commercial off-the-shelf products that may be transported by a drone or remotely controlled robot such as TALON, that can be used in a variety of hostile environments (see [8]), reducing potential dangers to the user.

Specifically, this paper presents the specification study of such a device, using Monte Carlo simulations to investigate the efficiency of different scintillator/detector materials: hyperpure-Ge (HPGe), lanthanum(III) bromide (LaBr) and thallium activated sodium iodide (NaI(Tl)), when detecting a wood or steel encasement target that has been buried in sand at depths of 0, 5 and 10 cm beneath the surface. The Monte Carlo experiments were performed using a SUN quad-core AMD Opteron Processor 8356 (16 processors) with 128 Gb of RAM memory, located at the University of Surrey.

## Methods

In carrying out simulations, the primary need has been to seek unambiguous target-related data in the presence of statistical fluctuations, militating against false events (positive or negative). In preliminary testing of initial conditions, this has pointed to use of emitted fluxes of ~ 10^9^ photons, a situation entirely practicable using existing betatron technology, e.g. a transportable/portable betatron from JME Ltd, Lowestoft, UK [9]. Tracking 10^9^ histories involves computational times per run of up to 10 hours, but is considerably exacerbated in simulations involving realistic detector response. To enable performance of the range of simulations desired, idealised spectral responses were obtained for the various situations described herein, subsequently convolved with the corresponding analytical description of detector energy response, using Matlab-based software developed in-house.

The betatron spectrum was modelled as a polychromatic pencil beam by sampling a PDF based on published betatron spectral data [10]. A metal filter was used to reduce the low energy component with respect to the annihilation peak. Section 2.2 describes how the filter thickness was determined.

Following filtration, the hardened X-ray beam was used to irradiate the target. In this study, the target was assumed to either be made of medium density wood encasement or mild steel for comparison. The overlying material was assumed to be homogenous sand. The target was displaced at three different depths in the sand (0, 5, 10 cm) and the detector was laterally displaced (offset) by 0, 10 and 25 cm. This is further discussed in section 2.5.

To reduce execution time in this study, Monte Carlo simulation was used to model the intrinsic backscattered photon flux generated in the irradiation process followed by an analytical model of the detector response. The intrinsic backscatter spectra were then attenuated, using the Beer-Lambert law, and statistically blurred to simulate the spectral uncertainty when using different detector materials. Three detector materials were considered in this study: NaI(Tl), LaBr and hyperpure Germanium (HPGe). Whilst HPGe is a direct detection technology, the other materials are scintillators that will require coupling to a photon-counting detector such as a photomultiplier tube. This is accounted for in the section 2.3.3.

To model each detector, the amount of statistical uncertainty (blurring) at each photon energy was modelled from published data as discussed in Section 2.1.1. The basic geometrical arrangement is described in Section 2.2. The various geometrical variations in target depth and geometric detector offset are investigated and presented with intrinsic backscattered spectra in section 3. The energy spectra are presented in Section 3 for each detector material. Section 4 discusses the results found and conclusions to be drawn.

### Betatron Source

**Fig 1:**
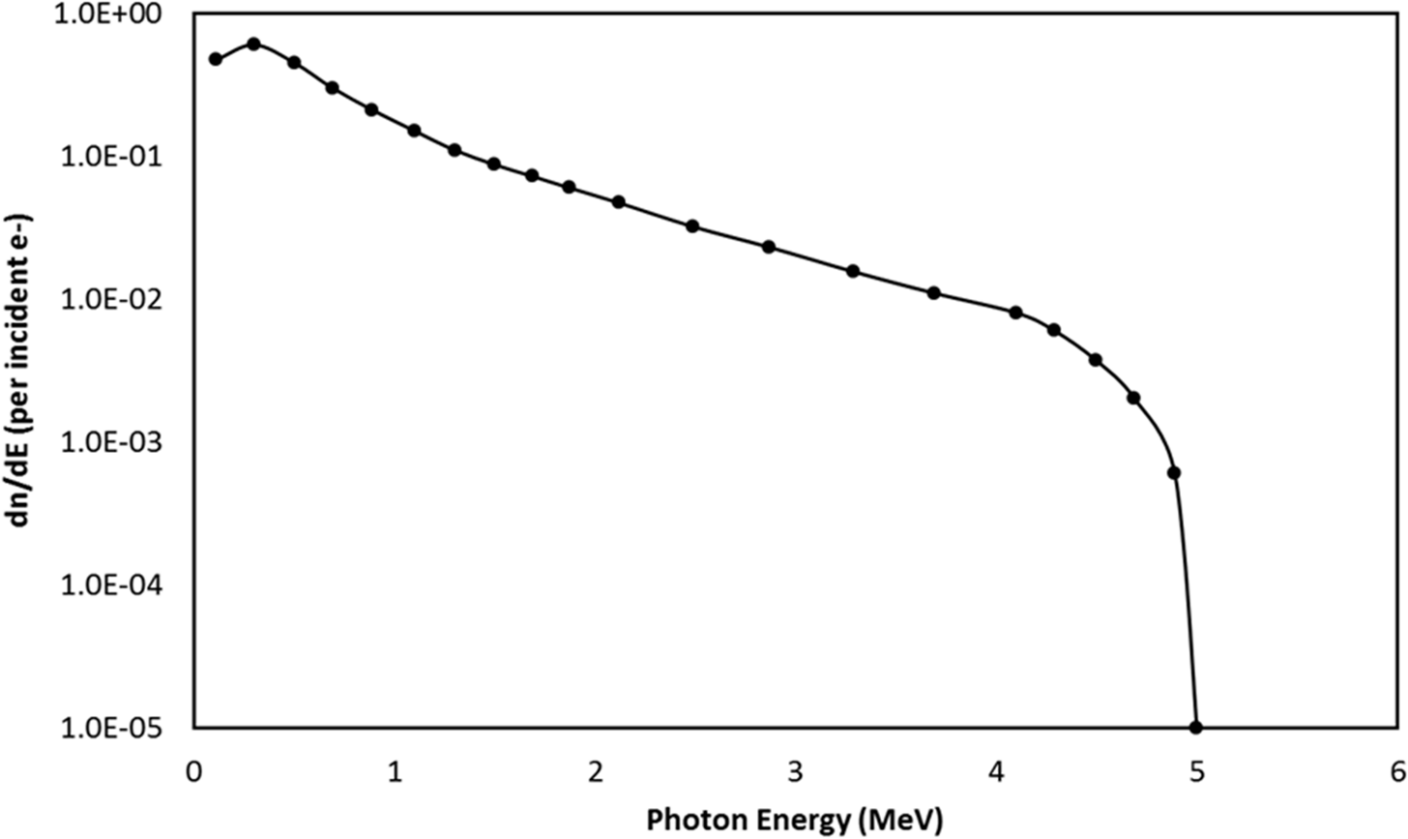
Bremsstrahlung x-ray (photon) energy spectrum from a 5 MeV electron beam. The coordinate (vertical axis), dn/dE, shows the number of x-rays within a certain energy range per incident 5-MeV electron. Adapted from source [10].

The bremmstrahlung X-ray spectrum of the betatron was implemented by sampling a PDF derived from data found [1], as shown in Fig 1.

**Fig 2:**
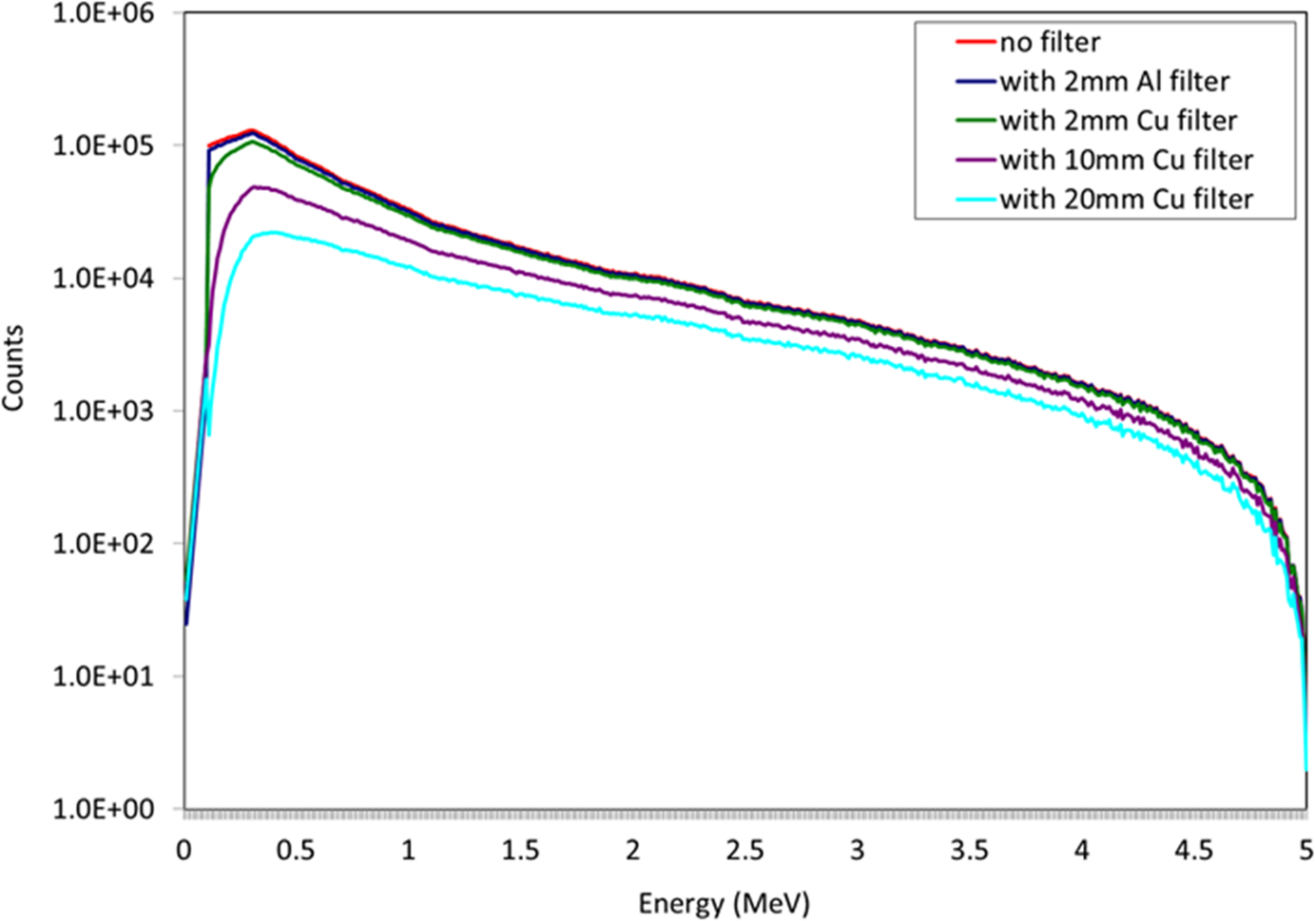
5 MeV energy spectra obtained at the exit of the betatron with and without the copper or aluminium filter. 1x10^7^ photons were simulated in each experiment.

A large number of low energy photons do not contribute to the final detection signal, therefore a filter was used to harden the beam exiting the source. Aluminium and copper filters were investigated to harden the beam with varying thicknesses. The results are shown below in Fig 2.

As can be seen, relatively little beam hardening was found when using 2 mm of Al. The Al filter was replaced with a Cu filter (shown in green in Fig 2) which was increased from 2 to 20 mm. At 20 mm, the hardened beam was somewhat flatter, compared to the unfiltered beam, with much of the unwanted low energy radiation attenuated. This was then adopted for all subsequent simulation experiments.

### Experimental Geometric Arrangement

**Fig 3:**
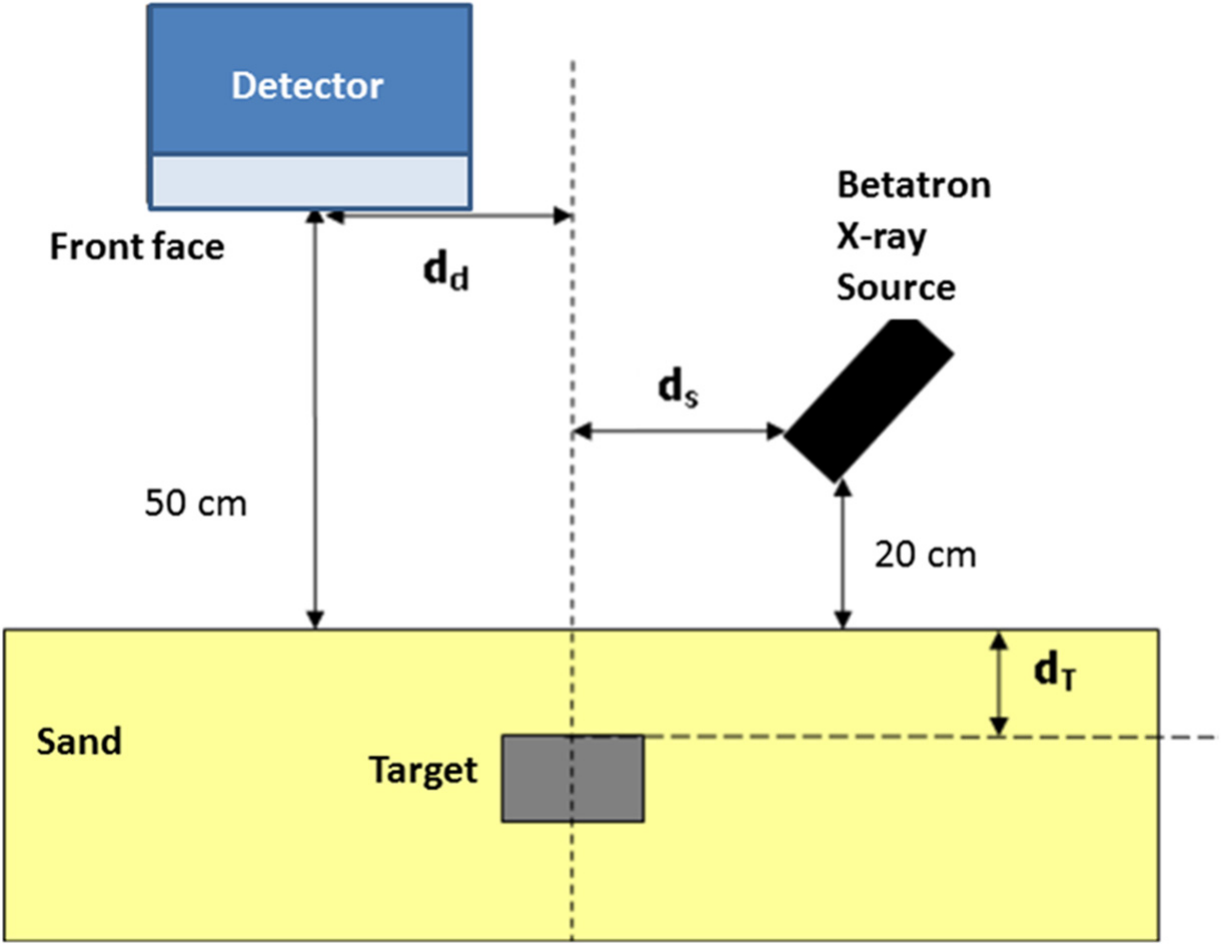
Experimental geometry used in X-ray backscattering simulation. Image not to scale.

The experimental geometry used in all subsequent Monte Carlo studies is shown in Fig 3 where d_d_ refers to the distance of the detector from the centre line (dashed). d_T_ refers to the distance of the top of the target from the sand surface. A standoff distance of 50 cm was chosen to produce a realistic representation of the energy spectra that would reach the detector, if mounted on the front of a remote-operated platform.

The source as discussed previously was modelled on a bremsstrahlung 5 MeV betatron energy spectrum. The X-ray beam was modelled as pencil beam passing through a 20 mm Cu filter before impinging on the target of size 10 cm (diameter) x 10 cm (length). It is worth noting that at 10 cm depth in sand with the aforementioned filtered source and geometry, approximately 60% of the normal incidence photons will have been attenuated. is explored in more detail in Section 3.

### Target Material

Two types of target are considered: wood and steel. Steel represents a common component in IEDs being the conventional material of choice for generating shrapnel or blast fragment materials. However, use of metal detectors in front-line operations has rendered this approach largely redundant so that wood is increasingly used as a cheap, lower density, but still potentially dangerous and difficult to detect fragmentation material. Whilst other materials may also be found in buried IEDs, these two materials represent some of the extreme target material that may be found within the IED detection sphere. As there are a number of steel compositions available commercially, mild steel composition was used in this study with a density of 7.89 g/cm^3^ [11]. Wood was modelled with a density between soft and hard wood (0.68 g/cm^3^) [12]. The composition of the two targets is shown in Table 1. It is considered unlikely that the constituent explosive chemicals would present an identifiable signature using this modality, although this aspect has not been fully explored in such contexts.

**Table 1:** Composition of the targets used in this study.

*lemental Proportion by weight (%)*			
*Medium Wood*		*Mild Steel*	
Hydrogen	6.0	Carbon	2.5
Carbon	50.0	Silicon	3.5
Nitrogen	0.2	Phosphorous	0.5
Oxygen	43.0	Sulphur	0.5
		Manganese	6.0
		Iron	98.7

**Fig 4:**
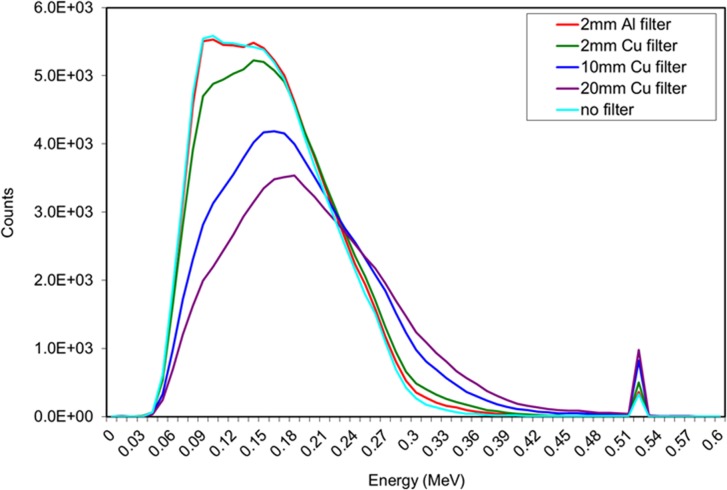
Backscattered spectra obtained with a steel target buried 5 cm into sand and the measuring plane (representative of the detector position) placed directly above the target with 50 cm standoff distance. Varying material and thickness of filter hardened the X-ray beam, resulting in reduced low energy backscatter and an enhanced annihilation peak.

A measuring plane was placed at the detector front face to measure the intrinsic backscattered spectrum from the target. Fig 4 illustrates the backscatter spectra obtained when the filter material and thickness was varied. It was decided that the 20 mm Cu filter provided the best spectrum, minimising the amount of low energy backscatter whilst increasing the relative height of the annihilation peak.

### Geometrical Variation

The effects of using different target materials, displacing the target within the sand at different depths and offsetting the detector from the principal axis of the target were investigated. The subsequent spectra were then attenuated by applying the Beer-Lambert law before being blurred with the energy response for the different detector materials.

### Burial Depth

In this experiment, the target is buried in sand at intervals of d_T_ = 0, 5, 10 cm and d_d_ = 0 cm (directly above the target). The intrinsic backscattered X-ray spectrum was scored at the front face of the detector plane for each set up. The effects of this were seen for wood and steel targets as shown in section 3.1.

### Detector offset

The detector was initially placed at 50 cm standoff distance from the surface of the sand. It was then horizontally translated by an offset distance, d_d_ of 10 and 25 cm, whilst maintaining the perpendicular standoff distance of 50 cm. The intrinsic efficiency in detecting the backscattered photons was calculated as the ratio of number of photons emitted to the number of photons detected in the set up shown in Fig 3. Table 2 shows the results of this preliminary experiment.

**Table 2:** The efficiency of detecting backscattered photons when the detector is moved from the centre of the target.

Distance between the detector to the centre of the target (d_d_)	Intrinsic Efficiency (%)
**0**	0.70 ± 0.03
**10**	0.56 ± 0.04
**25**	0.40 ± 0.04

### Detector Variation

In order to accurately model the observed pulse height distribution of the deposited energy spectrum, then the statistical variation in the photoelectron signal must be considered for any photon-counting approach. Whilst HPGe is a direct detection approach, we have assumed here that the remaining scintillator-based materials were coupled to a photomultiplier, thus producing an energy-dependent spectral blurring process representing the combined scintillator and photomultiplier detector configuration. This was achieved by using the well-known experimental model of energy-dependent peak broadening described by Knoll [13]. The energy resolution of a detector (measured as the Full Width Half Maximum, FWHM, divided by its peak amplitude) can be modelled as a function of energy [14]:

$$FWHM\;(keV)=\alpha E^{\left(1-\beta \right)}.$$ (1)

**Fig 5:**
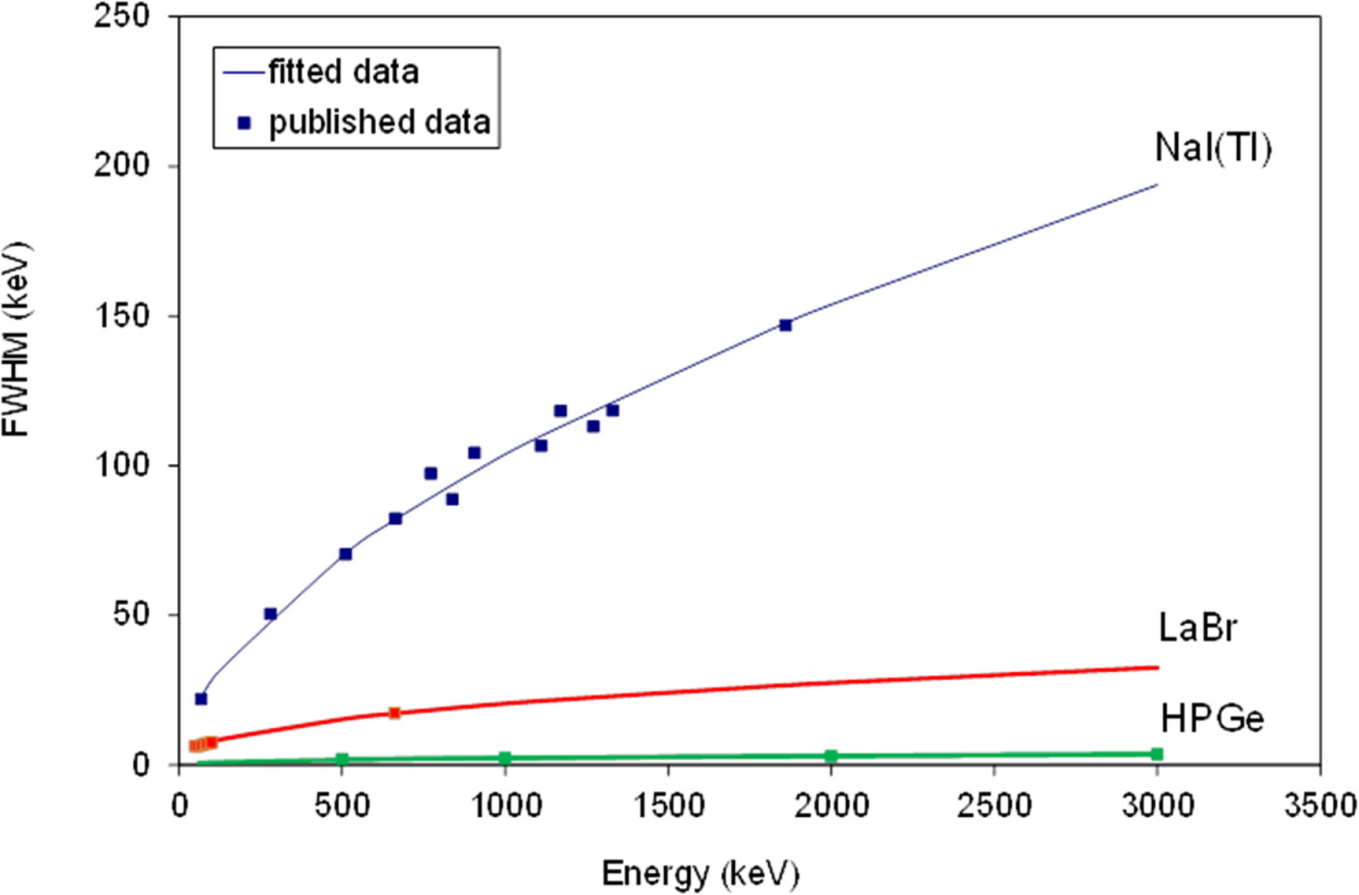
FWHM as a function of energy for NaI(Tl), LaBr and HPGe. These data have been fitted using Eq 1 and extended to the energy range of the betatrons to be used.

FWHM as a function of energy has been plotted in Fig 5 for different detectors. Table 3 shows the two parameters in Eq 1 fitted using Least Squares, for each detector. It should be noted that the ideal detector would produce a FWHM approaching a delta function, essentially independent of incident energy. Therefore, the ideal parameter fits would be α asymptotically approaching zero, and β approaching a value of unity. In this respect, the HPGe detector behaves in a manner close to that of such an ideal detector, although in practice, cost considerations may come into play. LaBr represents a good compromise candidate in terms of cost and performance, compared to NaI(Tl) which is the most ubiquitous scintillator in current use for radiation detection tasks.

**Table 3:** α and β coefficients fitted for each detector material shown in Fig 5.

Detector material	α	β	References
**NaI(Tl)**	2.02	0.43	[14]
**LaBr**	1.12	0.58	[16]–[19]
**HPGe**	0.127	0.58	[15]

The energy resolution has been modelled for NaI(Tl) from previous experiments [14]. HPGe has also been modelled from data published by Owens et al [15]. There is limited comprehensive data for LaBr as this is a relatively new detector materials in the field. Therefore, a number of publications have been collected to provide a wider energy range with which to fit the energy resolution model [15–19]. Plotting the results of these literature sources, and applying the model described in the equation above provided a good parametric fitting to the data.

## Results

### Intrinsic Backscatter Spectra with Burial Depth

**Fig 6:**
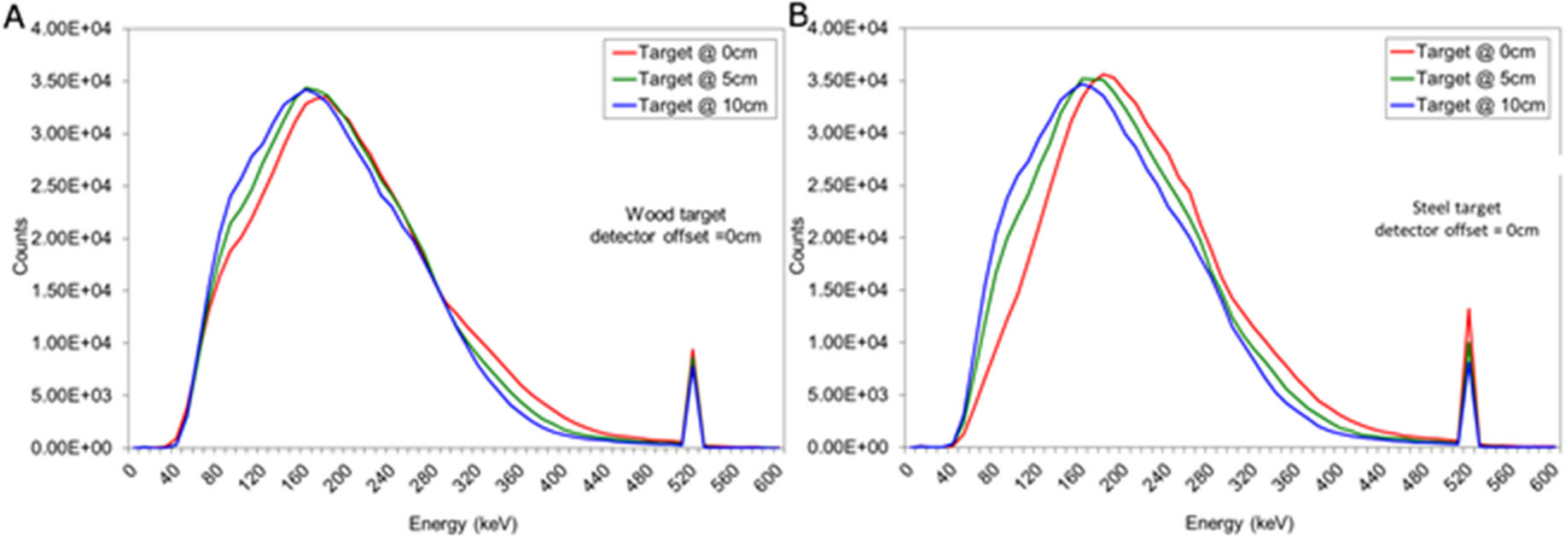
Backscattered spectra obtained w hen the (a) wood or (b) steel target was positioned at 0, 5 and 10 cm from the surface of the sand.

Backscatter spectra produced for wood and steel buried encasements are shown in Fig 6. These demonstrate a broad scatter peak and an annihilation photon peak. The wood target can be seen to deliver a small change in the low energy portion of the spectrum, whereas the steel target shows more pronounced effects, particularly with respect to the 511 keV peak with increasing depth.

As the target is moved to a greater burial depth from the surface of the sand (and the detector), there is a systematic loss of events from the photopeak intercepting the detector, due to well-known inverse square law processes, as well as further losses due to scattering in sand. Examining the bremsstrahlung-induced backscatter spectrum, there is also a loss of events seen at energies above the backscatter peak energy (160–180 keV), and a build-up of events at lower energies. This is also attributed to a build-up of Compton events scattering into the detector plane caused by overlying material coupled with the change in geometry. It is worth noting that any such change in geometry will also change the overall counting efficiency of the system.

**Fig 7:**
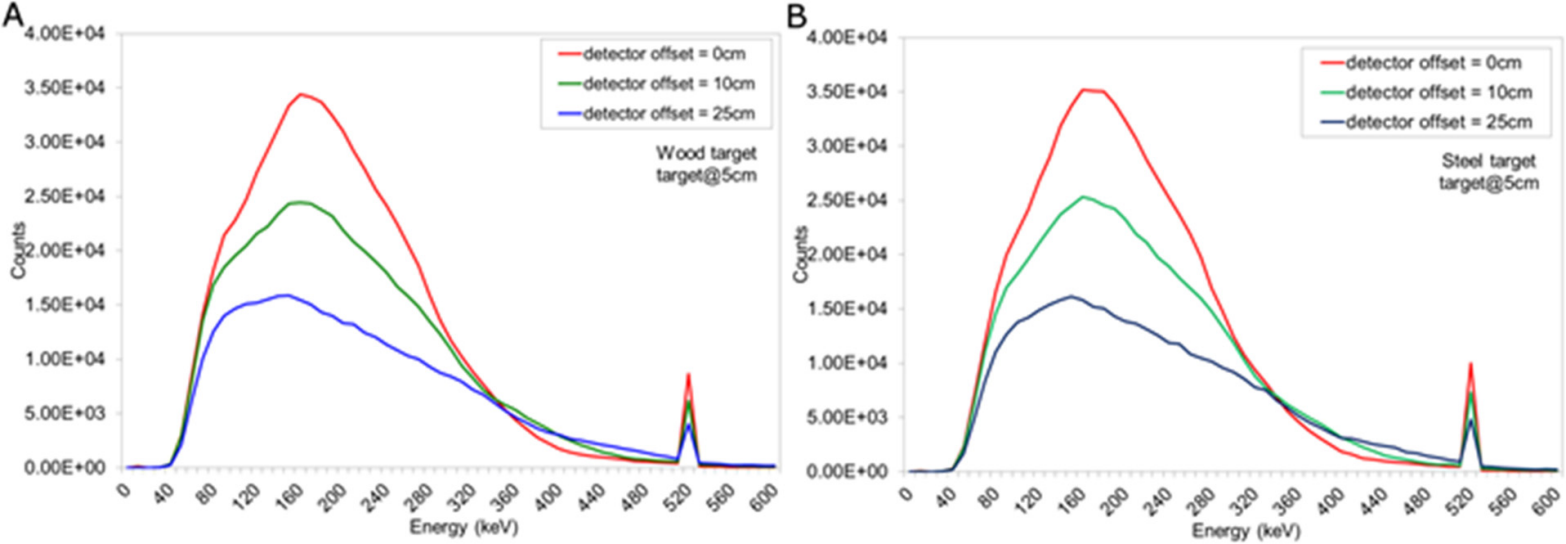
Energy spectra obtained when the a) wood or b) steel target was buried 5 cm in sand and the detector offset = 0, 10 and 25 cm.

In order to examine the differences in these spectra with background, then a series of Monte Carlo simulations were undertaken where the target volume was replaced by sand. We then produced difference spectra, as shown in Fig 7A) and 7B). This emulates the continuous background subtraction that would need to be produced for a vehicle deployed in theatre.

### Intrinsic Backscatter Spectra with Detector Offset

Fig 7 shows the spectra obtained when the detector was translated horizontally from the principal axis of the wood target. Shifting the offset of the detector reduces the event counts in the annihilation peak as well as the overall peak of the spectrum. As can be seen, the spectral shape remains the same whilst its magnitude reduces with increasing distance, d_d_. Shifting the offset of the detector reduces the event counts in the annihilation peak as well as the overall peak of the spectrum. Placing the detector directly above the target provides the largest solid angle with which to detect the backscattered photons.

### Detector Variation

After the intrinsic backscattered spectra were obtained, their energies were attenuated following the Beer-Lambert law as described in section 2 for each detector material. The spectra were then statistically blurred with the relevant detector energy response. This section examines the effect of using different detector materials.

### NaI(Tl)

**Fig 8:**
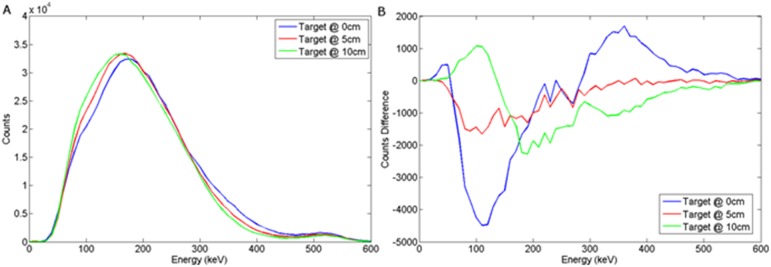
(a) Backscattered wood spectrum with d_d_ = 0 cm, d_T_ = 0, 5, 10 cm b) Subtracted wood to background spectrum for a NaI(Tl) detector.

**Fig 9:**
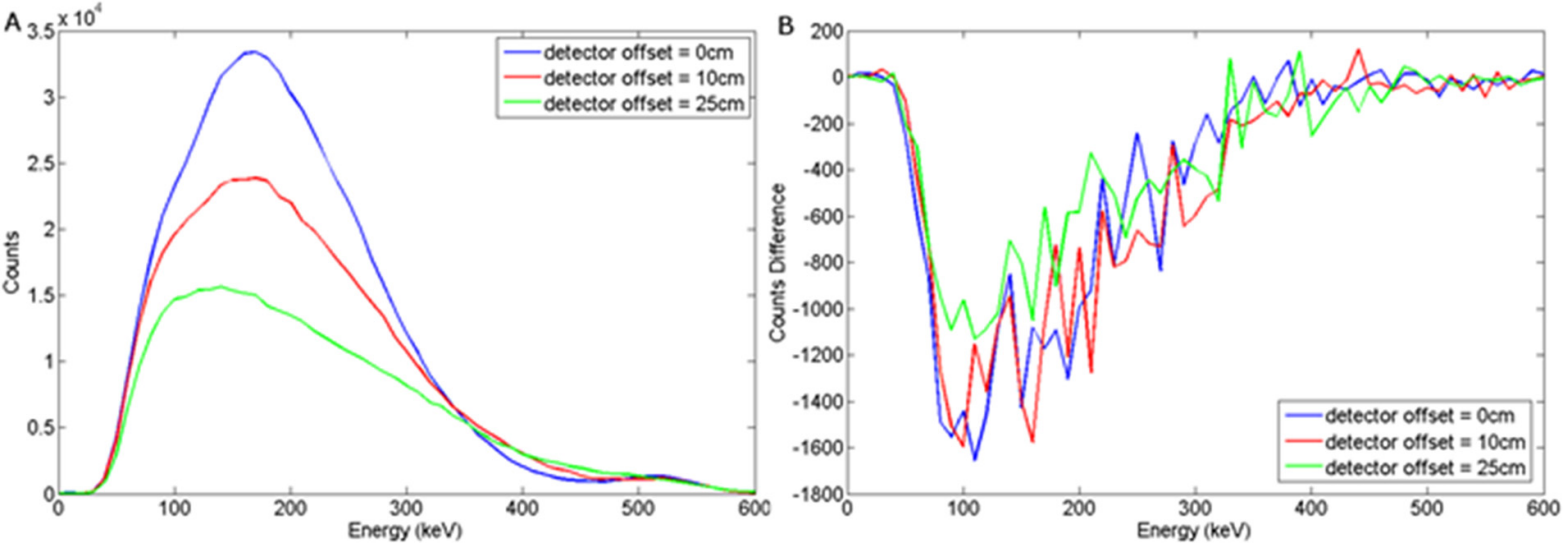
(a) Backscattered wood spectrum with d_d_ = 0, 10, 25 cm, d_T_ = 5 cm b) Subtracted wood to background spectrum for a NaI(Tl) detector.

**Fig 10:**
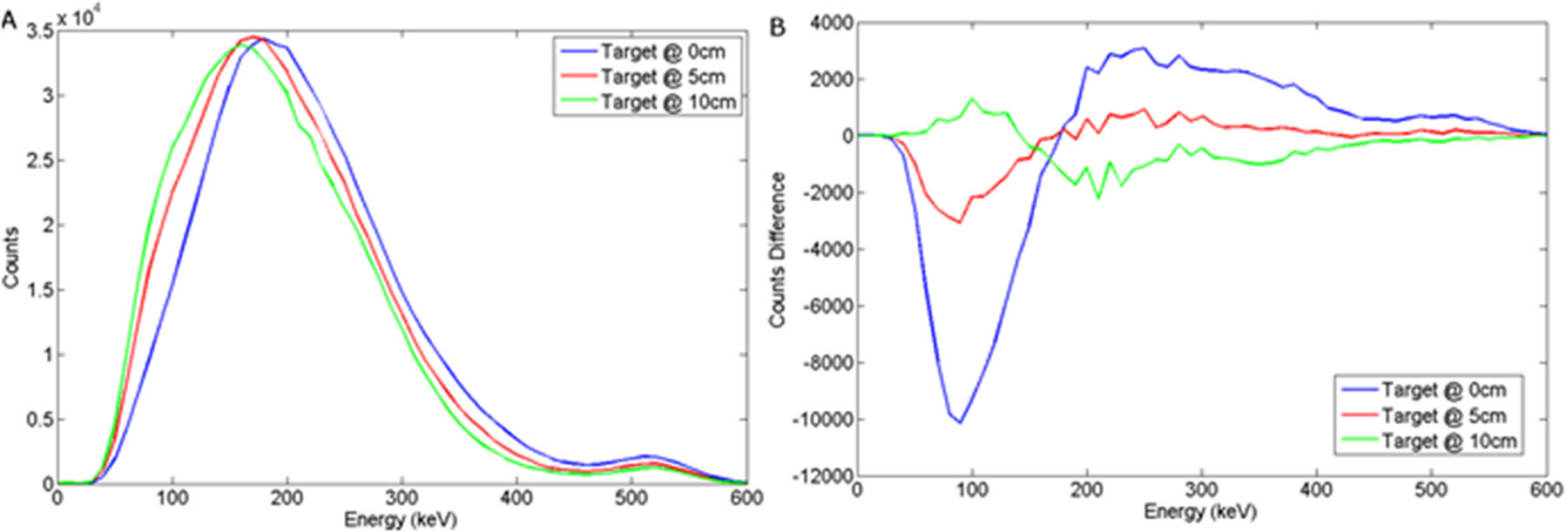
(a) Backscattered steel spectrum with d_d_ = 0 cm, d_T_ = 0, 5, 10 cm b) Subtracted steel to background spectrum for a NaI(Tl) detector.

**Fig 11:**
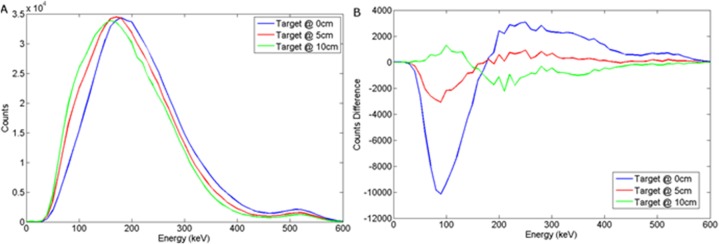
a) Backscattered steel spectrum with d_d_ = 0, 10, 25 cm, d_T_ = 5 cm b) Subtracted steel to background spectrum for a NaI(Tl) detector.

The graphs presented in Figs 8 and 9 illustrate the spectral response to a wood target in response to depth and offset variation respectively, when using a NaI(Tl) scintillator, coupled to a photomultiplier tube. For a steel target, Figs 10 and 11 show the dependency of spectral response using NaI(Tl) to variations in burial depth and detector offset respectively. Comparing Figs 8(A)–11(A) with the intrinsic spectra in Figs 6 and 7 we see very similar response, although the annihilation peak has been smoothed out due to photoelectron statistical broadening. Figs 8(B)–11(B) show the response when the sand background has been subtracted. In both cases, wood and steel casement targets, unipolar pulses are observed with varying tails as the source is displaced off-axis, whereas when the source increases in burial depth, we observe again unipolar pulse-like spectra but with tail-bound overshoot for surface targets. Figs 8B–11B suggest that the difference spectra between target and sand demonstrates little structural difference with lateral offset, showing a consistently behaved backscatter difference signal at 5 cm burial depth, but with some discernible differences between the difference spectra for wood and steel targets.

However, overall this demonstrates that a NaI(Tl) detector would probably be unsuitable in this application, as the annihilation peak may well be key to establishing depth and/or target information using signal analysis. Further analysis of this signal response has not been attempted here, but would make for an interesting subsequent study in its own right.

### LaBr

**Fig 12:**
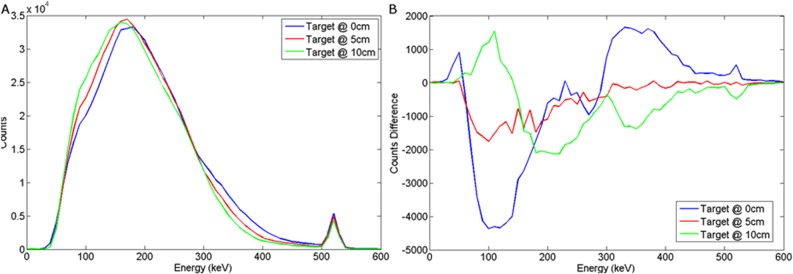
a) Backscattered wood spectrum with d_d_ = 0 cm, d_T_ = 0, 5, 10 cm b) Subtracted wood to background spectrum for a LaBr detector.

**Fig 13:**
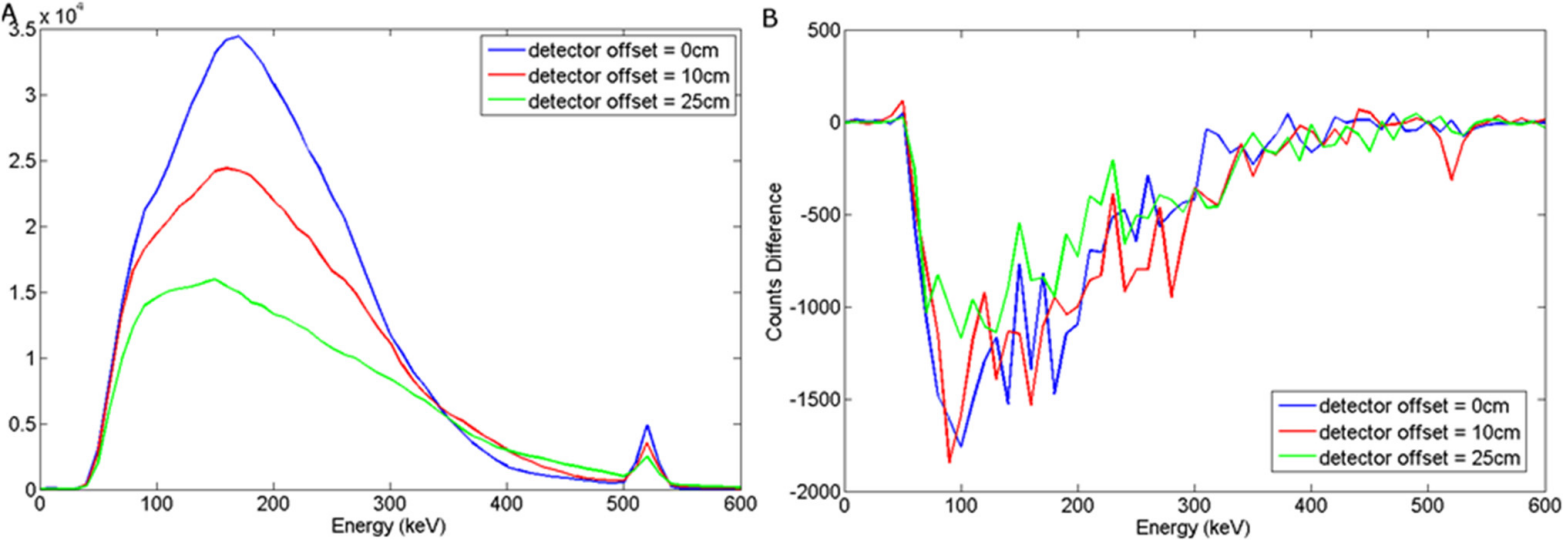
a) Backscattered wood spectrum with d_d_ = 0, 10, 25 cm, d_T_ = 5 cm b) Subtracted wood to background spectrum for a LaBr detector.

**Fig 14:**
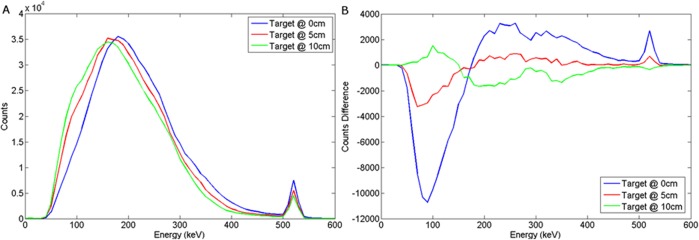
a) Backscattered steel spectrum with d_d_ = 0 cm, d_T_ = 0, 5, 10 cm b) Subtracted steel to background spectrum for a LaBr detector.

**Fig 15:**
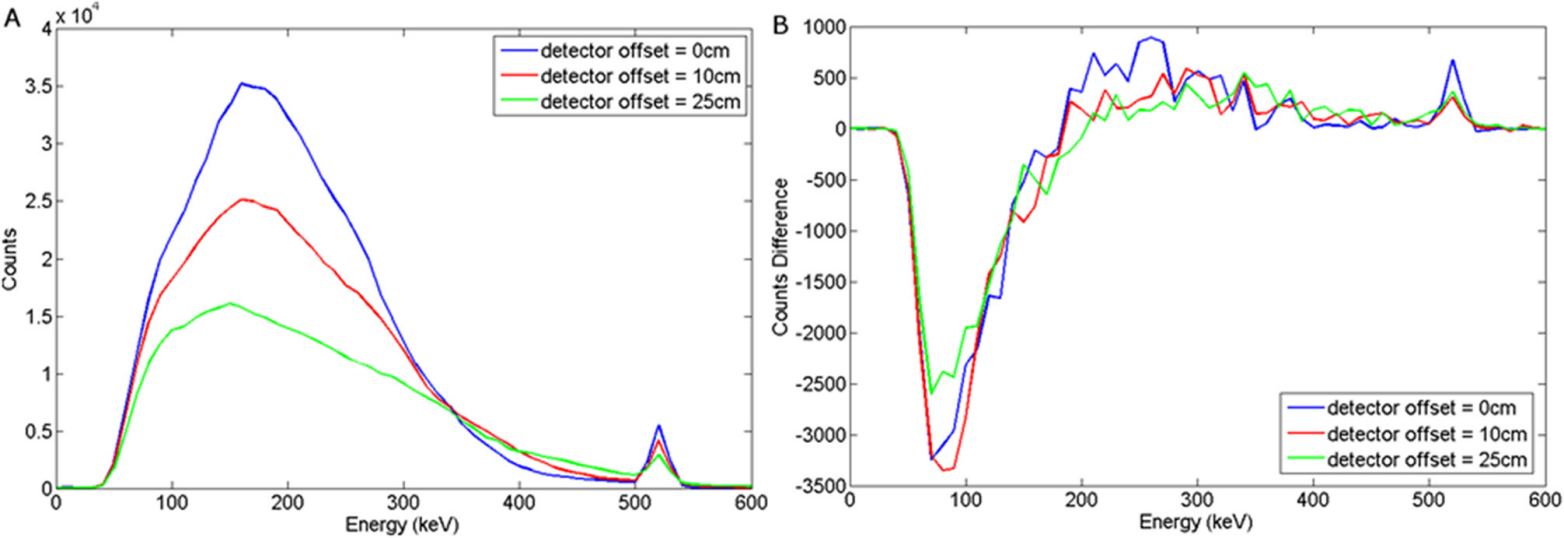
a) Backscattered steel spectrum with d_d_ = 0, 10, 25 cm, d_T_ = 5 cm b) Subtracted steel to background spectrum for a LaBr detector.

The spectral response using LaBr to variations in burial depth and detector offset for a wood target may be seen in Figs 12 and 13 respectively. For a steel target, Figs 14 and 15 show the spectral response using LaBr to variations in burial depth and detector offset respectively.

This exhibits a much better defined annihilation peak compared to NaI (Tl) which is attributed to the higher light output (per deposited unit of energy) with a density between that of HPGe and NaI(Tl), perhaps unsurprisingly yielding enhanced performance.

These spectra suggest that LaBr would be a good candidate detector material for this application.

### HPGe

**Fig 16:**
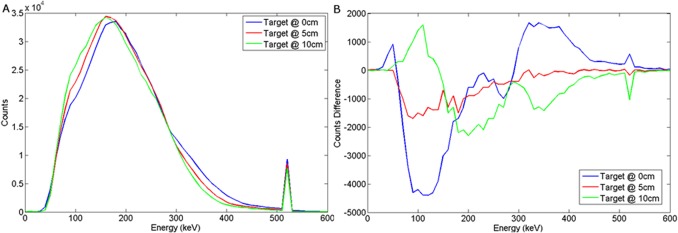
a) Backscattered wood spectrum with d_d_ = 0cm, d_T_ = 0, 5, 10 cm b) Subtracted wood to background spectrum for a HPGe detector.

**Fig 17:**
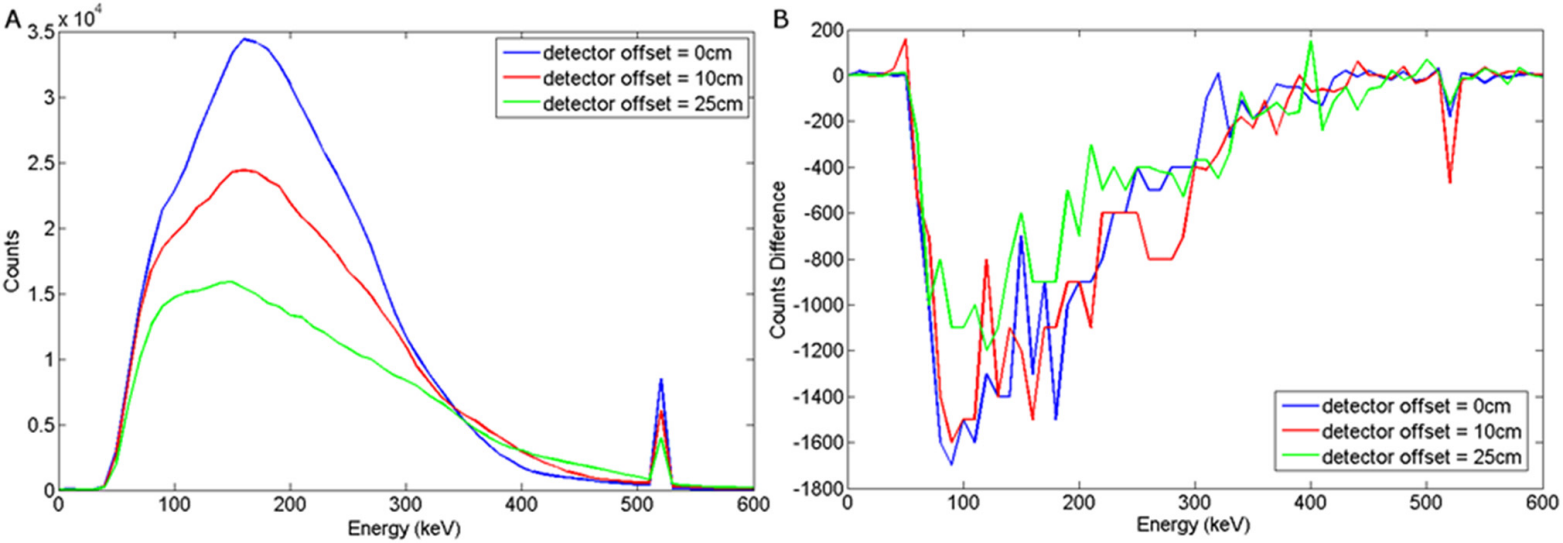
a) Backscattered wood spectrum with d_d_ = 0, 10, 25 cm, d_T_ = 5 cm b) Subtracted wood to background spectrum for a HPGe detector.

**Fig 18:**
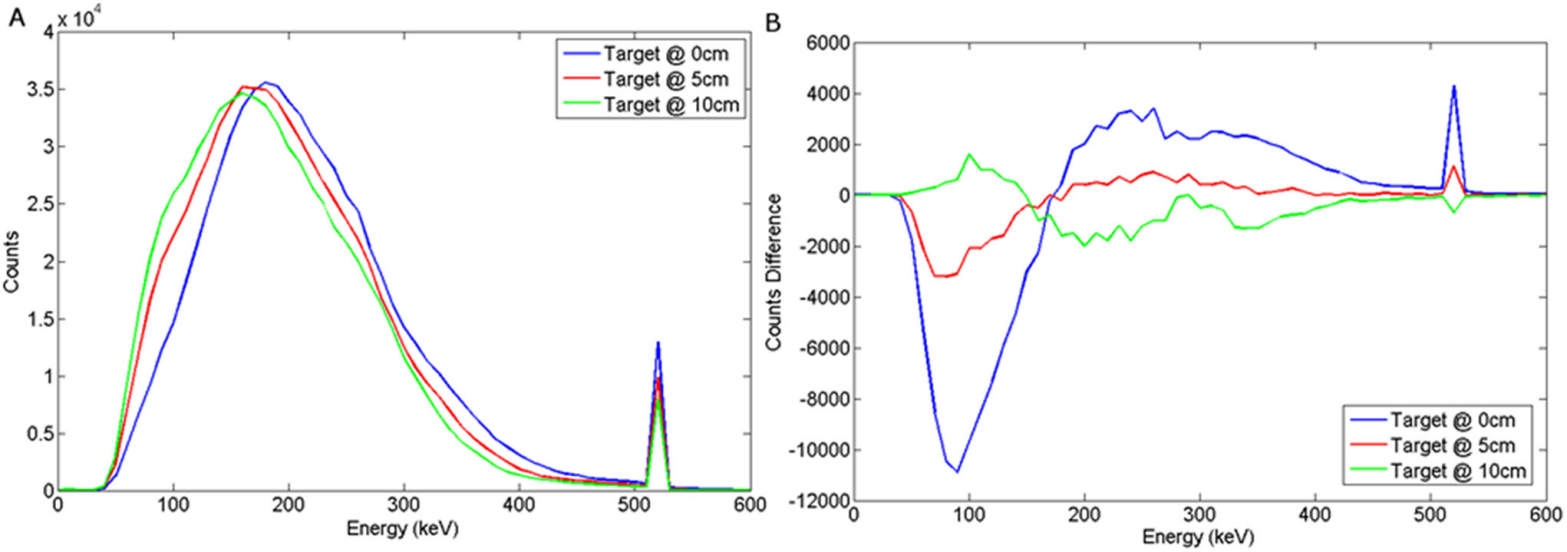
a) Backscattered steel spectrum with d_d_ = 0 cm, d_T_ = 0, 5, 10 cm b) Subtracted steel to background spectrum for a HPGe detector.

**Fig 19:**
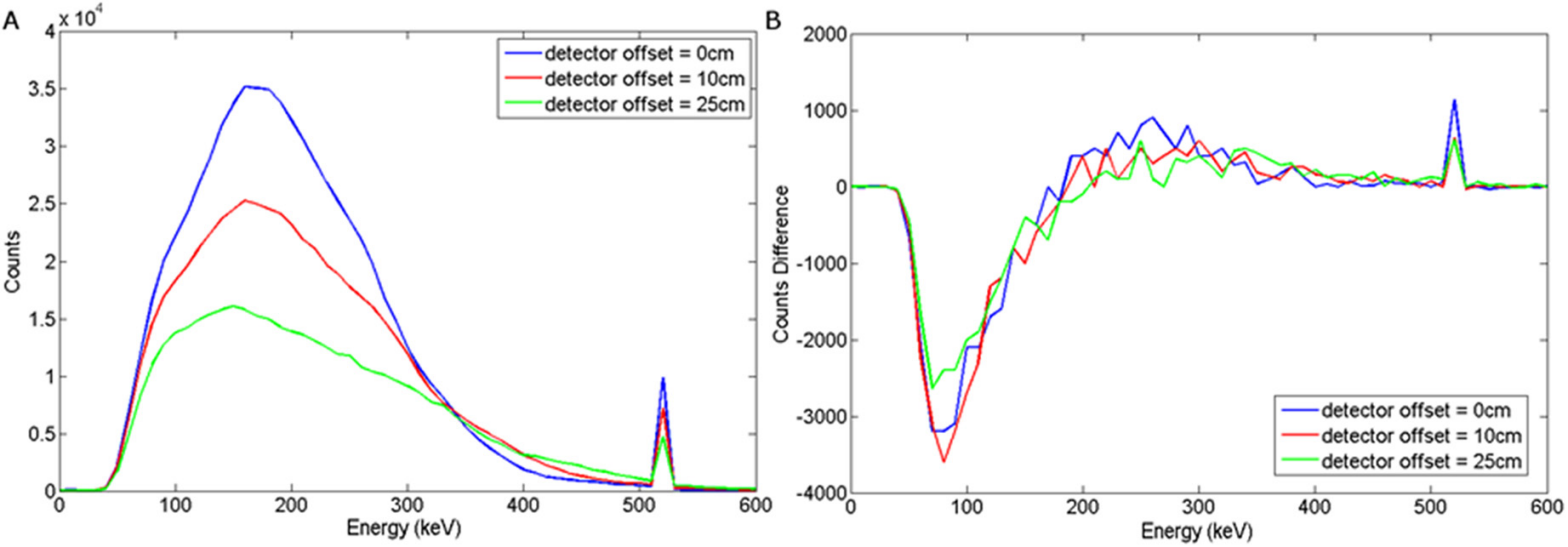
a) Backscattered steel spectrum with d_d_ = 0, 10, 25 cm, d_T_ = 5 cm b) Subtracted steel to background spectrum for a HPGe detector.

The spectral response of a cooled HPGe (Hyper Pure Germanium) detector to variations in burial depth and detector offset for a wood target may be seen in Figs 16 and 17 respectively. For a steel target, Figs 18 and 19 show the spectral response using HPGe to variations in burial depth and detector offset respectively.

This exhibits the clearest annihilation peak of all the detectors considered and also the best SNR in the subtracted spectra, particularly for a steel target. The unsurpassed energy response of HPGe coupled with its relatively high density results in almost ideal spectra for this application. The clear evidence of an annihilation peak present in observed and subtracted spectra could be harnessed to determine source depth and/or likely size/composition when compared to the broad backscatter component.

These results suggest that HPGe is an ideal detector candidate for this application.

## Discussion

The simulations herein have taken account of the dominant influencing factors in the use of a betatron source-target backscattered detection arrangement for buried IED detection. These factors include the emitted source spectrum and its filtration; wood and steel encasement targets buried at depths from superficial to 10 cm and use of a possible range of detectors, both scintillators and cooled solid-state detectors. It is apparent that use of a portable 5 MeV betatron and a LaBr scintillation counter provides one such realistic possibility for a workable system. Alternatively, HPGe could also be used in such an application.

The setup described could be installed in a threat survey vehicle that could be implemented with a portable betatron. This could be realised using either a single backscatter detector to probe a suspicious region or potentially using a linear array of collimated detectors to produce a real-time threat assessment over a prescribed route or path.

In terms of riders to the findings, the wood composition used in this study was a compromise for the large range of woods available in reality. As such the spectra derived here are only for guidance. Furthermore, given that the subtracted backscatter spectra were found to be somewhat equivocal at 10 cm burial depth, then incident fluxes greater than 10^8^ photons may be needed to reliably probe deeper targets. A further limitation of this study is that homogenous sand was assumed for the background of the target. However, in reality the background to the target would include rocks and other detritus which may provide a natural variation to the subtracted spectra. It should also be noted that false positives might also be triggered in encountering benign wood/steel objects or fragments at particular burial depths. The magnitude of such effects will impact on sensitivity and specificity performance and would be useful work for further study alongside assessing the ultimate minimum detectable target size/composition. This limitation may be eliminated by combining the spectral information with imaging techniques, or with the use of signature analysis using template matching [3]. However the use of advanced signal processing and pattern recognition methods is the subject of on-going and future work.

## Appendix

**Fig 20:**
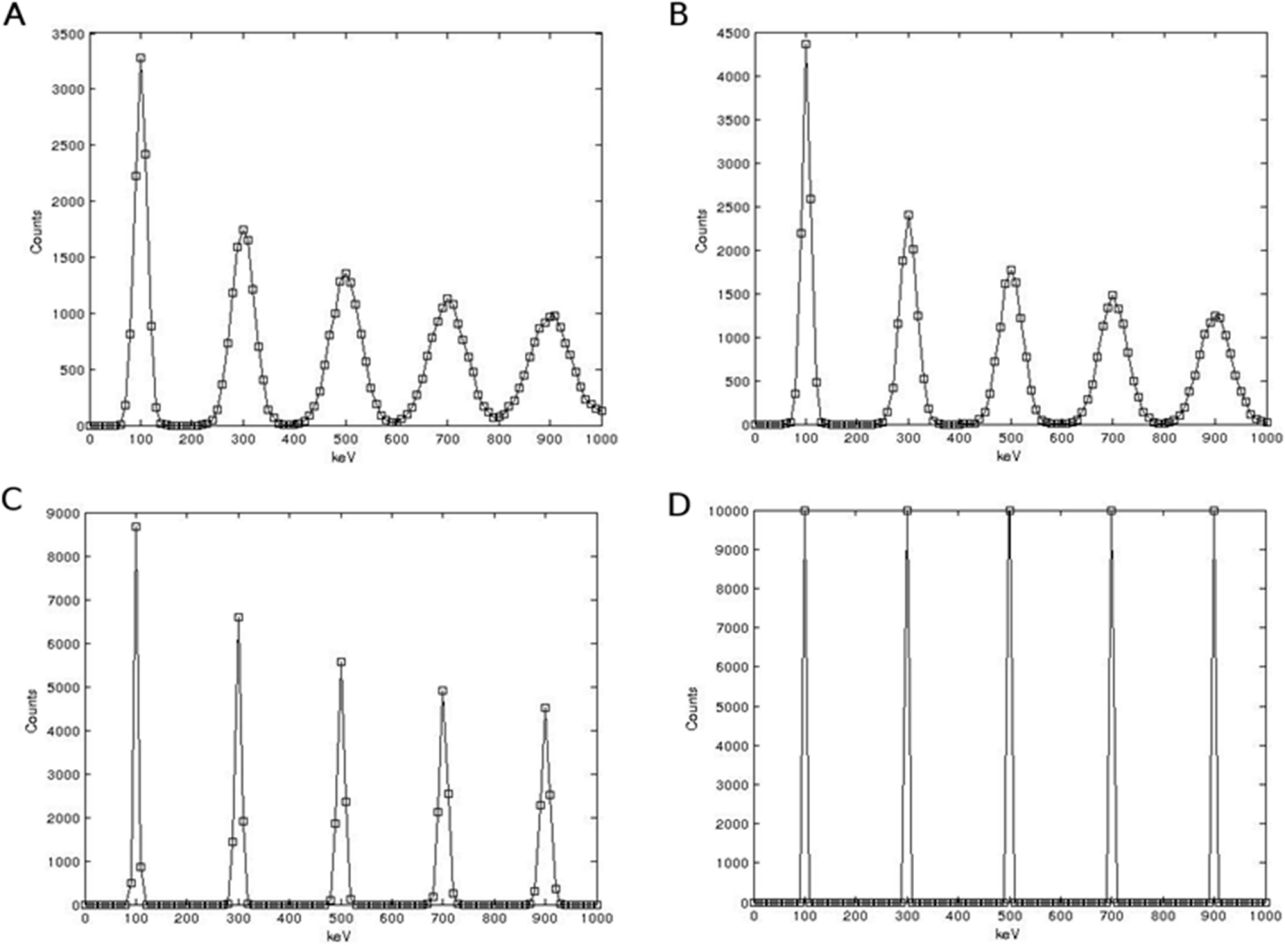
Blurred energy spectrum for (a) NaI(Tl), (b) LaBr, (c) HPGe.

Each detector’s energy response was validated with the use of an artificial energy spectrum. Delta spikes of 10000 events were generated at 200 keV intervals starting from 100 keV. The energy spectrum was then statistically blurred with the relevant detector energy response detailed in section 3. Fig 20(A)–20(C) show the results of this.

### Photon statistics

**Fig 21:**
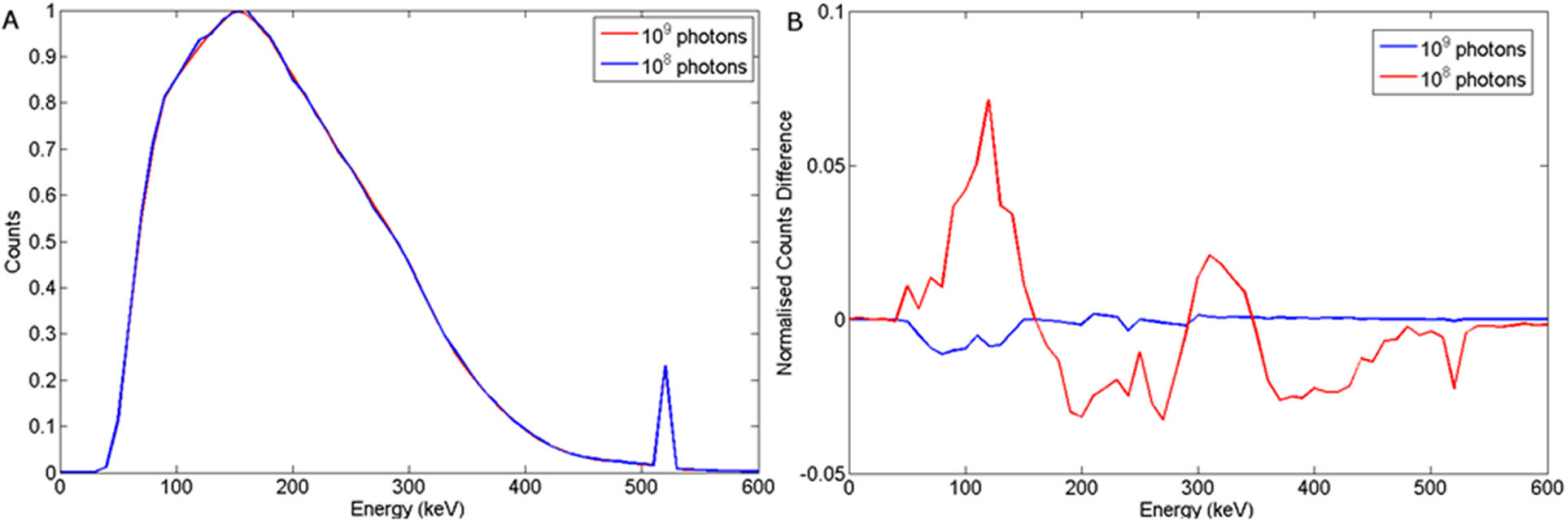
a) Normalised spectra obtained for a wood target with d_T_ = 10 cm and d_d_ = 10 cm when 10^8^ and 10^9^ photons were simulated. b) Corresponding normalised difference.

**Fig 22:**
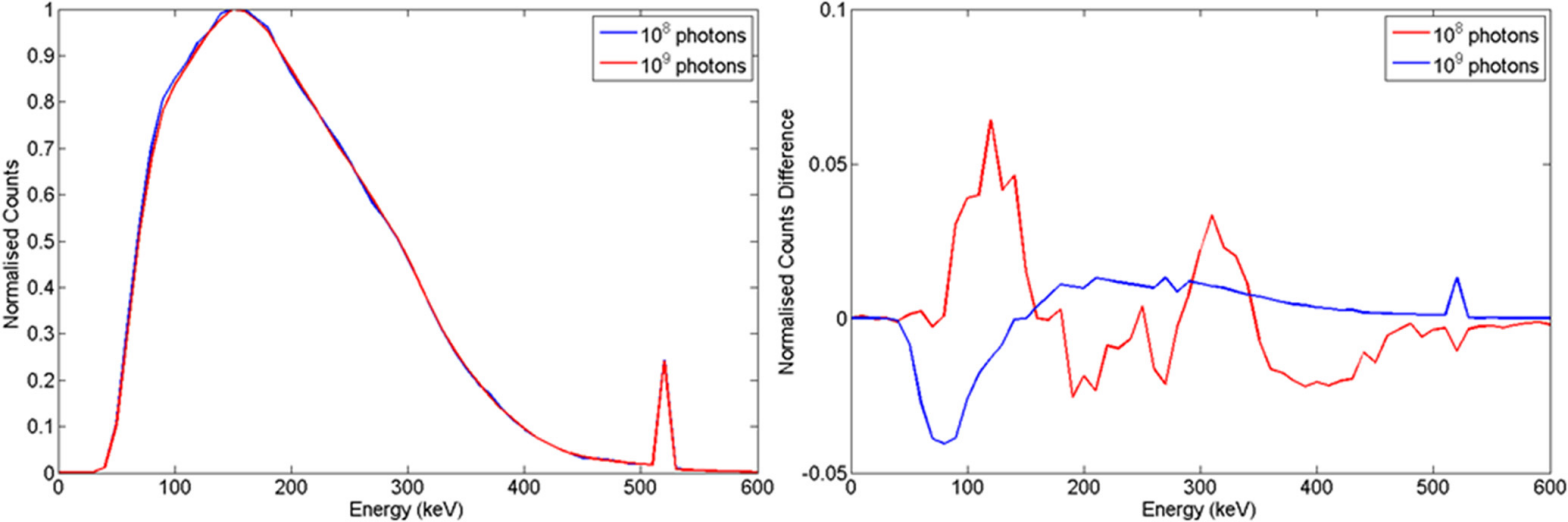
a) Normalised spectra obtained for a steel target with d_T_ = 10 cm and d_d_ = 10cm when 10^8^ and 10^9^ photons were simulated. b) Corresponding normalised difference.

This study has used 10^8^ photons throughout in its Monte Carlo simulations unless otherwise specified. It is likely that the betatron would emit more photons than this. To assess the accuracy of our assumption, a small experiment was carried out with 10^9^ photons simulated. Wood and steel targets were buried 10 cm into sand and irradiated with 10^9^ photons and a HPGe detector placed at 10 cm offset from the central axis (see Fig 3). Figs 21 and 22 show the results this experiment. As might be expected, increasing the photon statistics has little effect on the intrinsic spectra, but produces a difference spectra that yield useful structure, rather than statistical noise. Thus more challenging situations may require a somewhat higher flux than that simulated here.

## References

[1] Faust et al (2009), ‘Development of a Coded Aperture X-Ray Backscatter Imager for Explosive Device Detection’, IEEE Transactions on Nuclear Science. 56(1): 299–307.

[2] Datema et al (2002), ‘Experimental results and Monte Carlo simulations of a landmine localization device using the neutron backscattering method’, NIM Physics Research Section A: Accelerators, Spectrometers, Detectors and Associated Equipment. 488: 441–450.

[3] Bradley et al (2011), ‘Photon signature analysis using template matching’, Nuclear Instruments and Methods in Physics Research A. 652: 466–469.

[4] Buffler (2004), ‘Contraband detection with fast neutrons’, Radiation Physics and Chemistry. 71: 853–861.

[5] Chalmers (2003), ‘Single Sided x-ray inspection of vehicles using AS&E’s Z-Backscatter Van’, Proceedings of SPIE. 5199: 19–25.

[6] Dunn et al (2007), ‘Ionizing photon methods for standoff bomb detection’, Nuclear Instruments and Methods in Physics Research A. 580(1):778–781.

[7] Harding, Gilboy and Ulmer (1997), ‘Photon-induced positron annihilation radiation (PIPAR)—A novel gamma-ray imaging technique for radiographically dense materials’, Nuclear Instruments and Methods in Physics Research Section A. 398(2–3):409–422.

[8] Qinetiq (2009), ‘Fast, powerful and versatile, high payload robot technology. TALON’, Available from http://www.qinetiq.com/what/products/Documents/Talon-Robotics-Brochure-QinetiQ.pdf Accessed in September 2012.

[9] JME Ltd (2011), ‘Betatron Range’, Available from www.jme.co.uk/Betatron_Range.html Accessed in September 2012.

[10] Nuclear and Radiation Studies Board (NRSB) and Earth and Life Studies (DELS) (2008). Radiation Source Use and Replacement: Abbreviated Version. The National Academies Press pp67–84

[11] Suppliersonline (1999), ‘Carbon Steels 1010’, Available from www.suppliersonline.com/Research/Property/metals/809.asp Accessed in May 2011.

[12] Bradley et al (1991), ‘Photon Attenuation Studies on Tropical Hardwoods’, Appl. Radiat. Isot., 42(8):771–773.

[13] Knoll (2000), ‘Radiation Detection and Measurement’, Wiley publishings 3rd edition.

[14] Saripan 2006, ‘Design of a Wire-Mesh Collimator of Gamma Cameras’, University of Surrey PhD thesis.

[15] Owens et al (1985), ‘Spectral degradation effects in an 86cm^3^ (HP)Ge detector’, Nucl. Instr. Meth. A. 238:473–478.

[16] van Loef et al (2001), ‘High-energy-resolution scintillator:Ce3+ activated LaBr3’. Applied Physics Letters. 79(10):1573–1575.

[17] Menge et al (2007), ‘Performance of large lanthanum bromide scintillators’, Nucl. Instr. Meth. A. 579:6–10.

[18] Bousselham et al (2009), ‘Fano factor of scintillation detectors from photoelectron correlations’, IEEE Nuclear Science Symposium Conference Record. pp2370-2373.

[19] Khodyuk and Dorenbos (2010), ‘Non-proportional response of LaBr3:Ce and LaCl3:Ce scintillators to synchrotron x-ray irradiation’, J. Phys.: Condens. Matter. 22:485402. 21406744 10.1088/0953-8984/22/48/485402

